# New Drugs, Appliances, and Things Medical

**Published:** 1890-07-12

**Authors:** 


### NEW DRUGS, APPLIANCES, AND THINGS MEDICAL.

[All preparations, appliances, novelties, etc., of which a notice is desired, should be sent to The Editor, The Lodge, Porchester Square, W.]

### CALLARD'S DIABETIC FOODS.

We have received for examination varieties of the above, and subjoin the table of analysis:—

| | Sugar. | Starch. |
|---|---|---|
| 1. Gluten rolls ... ... ... | None. ... | Trace. |
| 2. Gluten cracknels ... ... | ,, ... | ,, |
| 3. Gluten rusks... ... ... | ,, ... | ,, |
| 4. Gluten and almond biscuits | ,, ... | ,, |
| 5. Bran biscuits ... ... | ,, ... | None. |
| 6. Almond biscuits ... ... | ,, ... | ,, |
| 7. Sponge cakes ... ... | ,, ... | ,, |
| 8. Sweet almond biscuits ... | ,, ... | ,, |
| 9. Cocoanut biscuits ... ... | ,, ... | Trace. |

These are all well made, appetising in appearance, and, chemically speaking, most suitable as foods for the diabetic. Many of them are so pleasant to the taste that the public at large may and will eat them with pleasure. A few are sweetened with saccharin, but this is present in such small quantity as to be perfectly innocuous, and, further, its presence makes the foods much pleasanter to the taste. We congratulate the maker on having added a number of fresh forms to the too limited list to which diabetics are confined. The foods are pure, palatable, and prepossessing in appearance, and present a sufficient difference of form and flavour to enable the unfortunate diabetic to obtain the variety which is more than usually charming to this class of patient. We can most heartily recommend these foods, not only to the sufferers for whom they are primarily intended, but also to those who want to reduce their starch and sugar-containing foods on account of obesity.

### EUCALYPTUS OIL IN INFECTIOUS DISEASES.

We think that a short *résumé* of the more important points and facts in Mr. Curgenven's paper at the Epidemiological Society on the use of Eucalyptus oil in infective diseases will be of interest to our readers. They are as follows:—

1. From the experiments of various observers, as Koch, Widal, and Chauternisse, it was found that volatile, aromatic, and camphoraceous disinfectants, when mixed with FIXED oils, fats, vaseline, or alcohol up to the extent of 5 per cent., had no effect in destroying bacilli. On the other hand, the author stated from experience when these volatile forms of germ-destroying agents were mixed with VOLATILE oils they retained their powers of disinfection.

2. The method of inunction in scarlet fever, introduced by Dr. Budd, had failed because of the unsuitableness of the agent applied. Fixed oils and fats also became rancid and offensive when extensively used for inunction.

3. The author condemned the use of all poisonous disinfectants (*a*) because dangerous *per se* to the patients; (*b*) because they required skilled attendants to apply them. Eucalyptus is entirely non-poisonous, and anyone can apply it. The solution he uses is a proprietary article, "Tucker's Eucalyptus Disinfectant. It is practically a solution in eucalyptus oil of thymol and other camphoraceous bodies. Internally he uses oleum eucalypti in 1-4 mimim doses every four hours. The air of the room is kept saturated, as far as possible, with the vapour of the drug.

4. The most remarkable point in the paper is the series of cases in which the throat symptoms were urgent, and there were other well-marked scarlatinal signs present, all of which yielded to the treatment within 48 hours. If a further experience of this method of disinfection bears out Mr. Curgenven's results a most valuable agent has been added to our list of curative drugs.

5. Mr. Curgenven not only uses the disinfectant on the actual patient, but advises as a precautionary method that all children liable to an attack of the disease, who have come into contact with the patient, should be well anointed. He gives several instances of well-marked scarlet fever where children have slept with patients, and been in constant and close intercourse with them, and yet after being anointed have not had the disease. This, of course, may be immunity such as we meet with in practice, but it is an immunity which is very unusual, for the number of cases the author cites is sufficient to exclude immunity as the sole condition preventing an attack; and the conclusion in favour of the eucalyptus treatment is very favourable, although, of course, like all treatments, it may not be absolutely perfect.

6. The author also states that from his experience the treatment enables patients to get about again in a much shorter time than the usual six weeks which is insisted on as a quarantine period. This, again, if borne out by experience, is a very great gain, for the prolonged confinement in one or two rooms when practically well is one of the most irksome of the incidents, or we might almost call it "sequelæ," of the fever.

7. As the aromatic disinfectants are chiefly eliminated through the lungs and kidneys, the author draws attention to the possible destruction of germs and amelioration of symptoms in connection with these organs.

8. He believes also that the eucalyptus treatment has almost a specific action in preventing influenza. He makes his patient sleep in an atmosphere of the oil by sprinkling the sheets and pillows with it before going to bed, besides using it during the day. In connection with this point, we have been told by Australians that the oil, or rather the bruised leaves of the tree, if used constantly as an inhalant, will more certainly cut short an ordinary cold or influenza proper than any other method of so-called cure. This not from one or two people, but repeatedly from many, for we have made a point of asking the question of late.

In conclusion, we consider that Mr. Curgenven has given us a remarkable paper, based on much personal experience, and we hope that during the next few months we may have a number of observations from other workers in this field, extending and confirming our author's observations, and also a further series from himself.

### ON THE USE OF ANATTO IN COLOURING MILK.

Some time since we were consulted by sundry milk sellers as to the legality and advisability of using a solution of anatto in order to render the appearance of their milk richer, and so to increase its sale, or to satisfy the wants of customers who desire a better class of milk. We answered that morally there was no right to do so, and that it was also contravening the Sale of Foods and Drugs Act, Section VI., Div. I., because it was selling milk plus anatto to make it look richer, and not milk alone, which the purchaser expects. In the course of our investigations we had a conversation with a gentleman connected with the milk trade, and the following are among the answers he gave to our questions:—

Q. Is it a common practice in the trade to "colour" the milk? A. Yes; especially at seaside places, where the public expect "a rich country milk." Also to render the "special nursery milk" for infants richer in appearance. So frequent is the practice in the former particular, that I have known vendors obliged to colour their milk because their customers on returning from their holiday at the seaside have complained of the poorness of the milk. This was done, although the home milk was a very rich sample when analysed. Q. Is it a difficult thing to leave off when once begun? A. Yes; as complaints of "poorness" are at once made, though the milk is from the same cows. Q. How is the anatto obtained? A. From certain wholesale houses in London. A traveller calls and illustrates the improvement in appearance by adding some of the compound to the milk. Q. Is it not used very largely according to the statements of the vendors? A. Yes. It was stated to me to be used by eight out of ten milk sellers. Q. Does it tend to make the milk look better? A. Yes, more rich in colour; that is yellower, as if there were more cream in it. It also appears to me to make the milk thicker. Q. Have you known it to do harm? A. One case of an infant, after taking the milk, became poorly, and the doctor on being called in, said the milk did not agree. Q. How is the anatto used? A. A few drops of the concentrated solution sold by the agent are added to a small quantity of water, and this is added to a definite quantity of milk. Q. What reply did the agent make on being asked the question as to whether the solution is injurious or not? A. He stated the substance was harmless in the proportion used, and that no case of detection or prosecution had occurred from its use.

We also sent two samples of the same milk, but one of which was coloured by anatto, to Somerset House for analysis. The analyst stated in his report that the milk of which the samples were composed was of good quality. That one sample was coloured, and that the colouring matter was not injurious. The solution of anatto used is prepared from the cake or roll. Anatto is imported. Anatto is a vegetable colouring matter, and in the purest form is practically a semi-solid aqueous abstract from the fruit of the anatto plant. The solution is generally alcoholic and alkaline, and exhales a most fœtid and disgusting odour. There is on record the case of the death of an infant following a small dose of concentrated "colouring." It has been used for years to colour certain kinds of cheese.

As the result of our investigations, we feel constrained to state that the practice of using anatto in milk before sale is a breach of the Act of Parliament, it renders the salesman liable to prosecution, and, in all probability, must prove deleterious to many who drink milk so treated, and especially to young children. We understand that the anatto manufacturers make considerable profits by the trade, and we earnestly direct the attention of the medical officers of health and the sanitary authorities throughout the country to this objectionable practice, which ought to be promptly stopped by proceedings at law. In the meantime we warn the public that by choosing "rich country milk" they may unwittingly cause illness to members of their families, and, in any case, consume an article which is distinctly adulterated with a vile-smelling compound, which, in its concentrated state, must produce a sense of nausea and disgust. Unfortunately, it is difficult to detect anatto by analysis, but this difficulty can be easily overcome by those health authorities and their medical officers who use ordinary vigilance in the discharge of their public duties.

